# A comparative analysis of potential spatio-temporal access to palliative care services in two Canadian provinces

**DOI:** 10.1186/s12913-015-0909-x

**Published:** 2015-07-17

**Authors:** Nadine Schuurman, Ofer Amram, Valorie A. Crooks, Rory Johnston, Allison Williams

**Affiliations:** Department of Geography, Simon Fraser University, Burnaby, Canada; School of Geography and Earth Sciences, McMaster University, Hamilton, ON Canada

**Keywords:** Palliative care, Rural health, GIS, Health service access

## Abstract

**Background:**

Access to health services such as palliative care is determined not only by health policy but a number of legacies linked to geography and settlement patterns. We use GIS to calculate potential spatio-temporal access to palliative care services. In addition, we combine qualitative data with spatial analysis to develop a unique mixed-methods approach.

**Methods:**

Inpatient health care facilities with dedicated palliative care beds were sampled in two Canadian provinces: Newfoundland and Saskatchewan. We then calculated one-hour travel time catchments to palliative health services and extended the spatial model to integrate available beds as well as documented wait times.

**Results:**

26 facilities with dedicated palliative care beds in Newfoundland and 69 in Saskatchewan were identified. Spatial analysis of one-hour travel times and palliative beds per 100,000 population in each province showed distinctly different geographical patterns. In Saskatchewan, 96.7 % of the population living within a-1 h of drive to a designated palliative care bed. In Newfoundland, 93.2 % of the population aged 65+ were living within a-1 h of drive to a designated palliative care bed. However, when the relationship between wait time and bed availability was examined for each facility within these two provinces, the relationship was found to be weak in Newfoundland (R^2^ = 0.26) and virtually nonexistent in Saskatchewan (R^2^ = 0.01).

**Conclusions:**

Our spatial analysis shows that when wait times are incorporated as a way to understand potential *spatio-temporal* access to dedicated palliative care beds, as opposed to spatial access alone, the picture of access changes.

## Background

The provision of palliative care services is highly varied. This is true in multiple contexts, including differences in service provision between countries and also between jurisdictions within the same country [1, 2]. For example, a recent study examining the evolution of hospice palliative care in Canada concluded that the varied nature of funding structures, planning processes, and regionalization processes across the country’s provinces and territories have resulted in significant differences in what constitutes palliative care and how it is delivered both within and between them [3]. Clearly in a large and geographical diverse setting, such as Canada, there will be differences in service provision [4, 5]. We are particularly interested in how those variations in services affect access. This is challenging for a few reasons, including because what constitutes access can be thought of in multiple ways.

‘Access’ to health services is a complex concept, and there is no single way to understand what constitutes good or reasonable access to health care [6, 7]. For instance, smaller, non-tertiary facilities may report “flex” palliative beds that may or may not be available at any given time. Despite the complexity of assessing access – given a myriad constraints, it is essential that we understand the factors that serve to facilitate or block patients’ access to needed health services, including palliative care, in order to mitigate, minimize, or eliminate barriers [8]. The development of such an understanding can lead to informed or evidence-based decision-making [9–11]. At the same time, developing this type of understanding requires having analyses or data that policy makers and health system administrators can use to inform their understandings of barriers and facilitators to access [12–15].

In this article we present new methods designed to examine potential spatio-temporal access to facilities with dedicated palliative care beds in Canada. We run these methods using the case of two Canadian provinces: Newfoundland and Saskatchewan. By examining potential access it means that we are focused not on actual service utilization but on population-level factors that can inform service use [16]. There is little research examining potential spatial access to any form of palliative care service, though there are some exceptions [17–19]. Further to this, our inclusion of *both* spatial and temporal variables is novel and creates a nuanced portrait of what potential spatial access to facilities with dedicated palliative care beds actually looks like on the ground through the maps that are produced.

## Methods

### Data and methods

The methods section focuses on the development of the geographic information systems methods and tools used to enable description of the one-hour travel time catchments around palliative care services (PCS). We defined palliative care services as inpatient medical facilities that reported having at least one bed prioritized for or dedicated to the provision of palliative care to capture the on-the-ground palliative practices at play instead of relying on top-level funding categorizations for beds.

The one-hour catchments refer to the distance on a road network around the PCS that is accessible by automobile travel within one hour. We use figures in several cases to illustrate methodological concepts and procedures.

Newfoundland and Saskatchewan were selected as two test sites for three reasons: (i) they are geographically and culturally distinct and distant from each other whilst remaining part of Canada; (ii) they are products of different provincial health care systems; and (iii) health authorities across both provinces were willing to participate in this study.

**Fig. 1:**
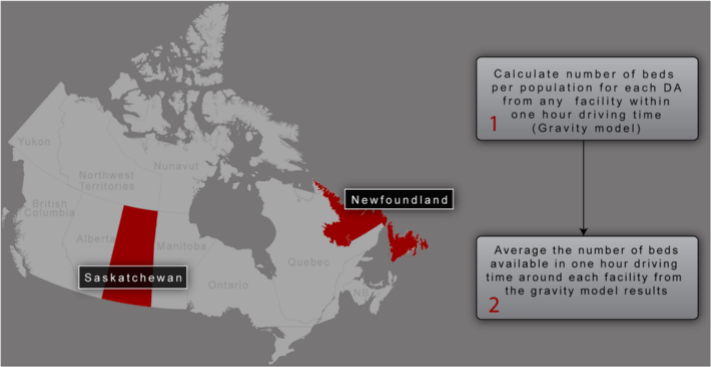
Illustrates the spatial context of the provinces of Saskatchewan and Newfoundland in the country of Canada. It also provides a simple explanation of the method used to calculate beds per catchment

The data collection and protocol were simplified into a single flowchart style graphic illustrated below in an overview graphic (Fig. 1). As depicted, Census data including population and road networks were combined with survey data from inpatient health care facilities in the two provinces to determine *where* palliative facilities were located and to calculate travel times to each facility. We then used the survey data to determine exactly what types of services were offered in each facility as well as number of beds and wait times. Using these simple variables (denominator populations, road network data, facility locations, beds per facility and wait times), we were able to construct detailed pictures (Figs. 4 and 5) of the actual wait time and access situation in each of the two provinces. A detailed methods description follows.

#### Data utilization and collection

Two datasets, DMTI Route Logistic data and Canadian population census data, were used to create the one-hour road travel time catchments around each palliative care facility. The DMTI dataset provided road network driving time estimations based on speed limit and road segment length. As the DMTI data is Canada-wide, the same dataset could be used for both provinces of focus.

Following approval from our Office of Research Ethics (at Simon Fraser University) and ten regional health authority ethics boards, we conducted a telephone survey of all inpatient health care facilities in Saskatchewan and Newfoundland. Saskatchewan has 11 distinct regional health authorities.; nine of them requested that we apply for ethics approval – which we did successfully. In Newfoundland, one health authority requested ethics approval – for which we complied. The names of the Ethics boards from which we received approval in Saskatchewan are as follows: Cypress Health Region; Regina Qu’Appelle Health Region; Kelsey Trail Health Region; Northern Population Health Unit (includes 3 regions); Prince Albert Parkland Health Region; Sunrise Health Region; Heartland Health Region and Prairie North Health Region. In Newfoundland, we received ethics from the Central Health Authority.

The phone surveys were conducted to determine the numbers of dedicated palliative care beds^1^ in each and their locations. Phone surveys were conducted with a knowledgeable staff member in each facility by the fourth author over a four-month period. After the study information was shared and verbal consent was obtained, respondents were asked about the numbers of dedicated palliative beds in the facility, the on-site services available for palliative care recipients, the average wait times for dedicated palliative care beds, and were asked to rank the relative availability of beds-to-patients using a five-point Likert scale. The Likert scale asked respondents to characterize the availability of designated palliative care beds in (your) hospital when they are needed with choices ranging from “designated beds are never available for new patients (i.e. a waiting list is always used)” to “designated palliative beds are always available for new patients (i.e. patients are never places on a waiting list for designated palliative beds).” Responses were entered directly into a spreadsheet during the survey process. Each survey took approximately five minutes to complete. In addition, we confirmed the facility addresses for geocoding purposes.

The address information for each facility was then geocoded (i.e. assigned a map location). Two datasets, DMTI Route Logistic data and population census data, were used to create one-hour road travel time catchments around each palliative care facility. The ‘population dataset’ was obtained from the 2011 Canadian census collected by Statistics Canada. Population variables (namely the number of residents) were gathered from the census at the dissemination area (DA) level. Three variables were used: total population; total population over 65; and total population over 85. Each DA is typically composed of neighboring streets that host between 400 and 700 residents. Because of differences in population density across geographically diverse communities, DA-level data is highly accurate in urban areas but is less so in more rural settings – where DA geographic size is generally less compact – and includes larger tracts of land but with the same average population per unit.

### Spatial analysis methods

The methodology used in this study builds upon that used by Schuurman et.al in an analysis of community accessibility to palliative care services in the Canadian province of British Columbia conducted in 2009 [17]. For this study, we used the ODMatrix function provided within the Network analyst extension in ArcGIS to calculate the driving time from each origin, or each facility with designated palliative care beds, to each destination, or DA centroid, along the network. This permitted the creation of a table with driving times from each facility to each of the DA centroids. We then included only those DAs within a one-hour road travel time in the catchments. In order to ensure that we included homes and small communities that were not directly on a major highway or road, we used a tolerance value of 2500 m to ensure that close to 100 % of the population in any DA was included in the count. All people residing within 2500 m of a major road were included in the population counts.

### Accounting for rural areas

In rural areas, where DAs are very large in size, the application of a tolerance value of 2500 to the ODmatrix was problematic in that it did always not capture the DA centroid. In such cases, the analysis may indicate that the population within the DA is outside the one hour catchment when, in reality, the population is within the catchment. In order to overcome this issue, we used the ‘calculate service area’ function within the network analyst to produce a one-hour polygon catchment around each palliative facility. We then created maps illustrating catchments that include rural populations on roads within a one-hour travel time of the facility.

Next, a series of analyses were implemented that contrast the availability of dedicated palliative care beds in facilities to the average reported wait time for these beds. As noted above, the average wait time for beds in each facility was collected during the survey. Bed availability was calculated using a gravity model (also known as a two-step floating catchment), wherein a 60 min driving time catchment was created around each facility. The bed-per-population ratio was then calculated for each facility by summing the population of all DA’s within the catchment. A 60 min catchment was then calculated around each DA in order to identify all facilities within the catchment and to calculate the total beds available [20]. Bed availability was then recalculated by averaging the rate of availability within the 60 min catchment for each facility. Finally, the relationship between wait time and bed availability was analyzed for each facility.

## Results

The survey process identified 26 facilities with dedicated palliative care beds in Newfoundland and 69 in Saskatchewan. Spatial analysis of one-hour travel times and palliative beds per 100,000 population in each of the two provinces show distinctly different geographical patterns. Local topography and road networks, settlement patterns and differing legacies of health care allocation potentially explain these very different patterns of access.

**Fig. 2:**
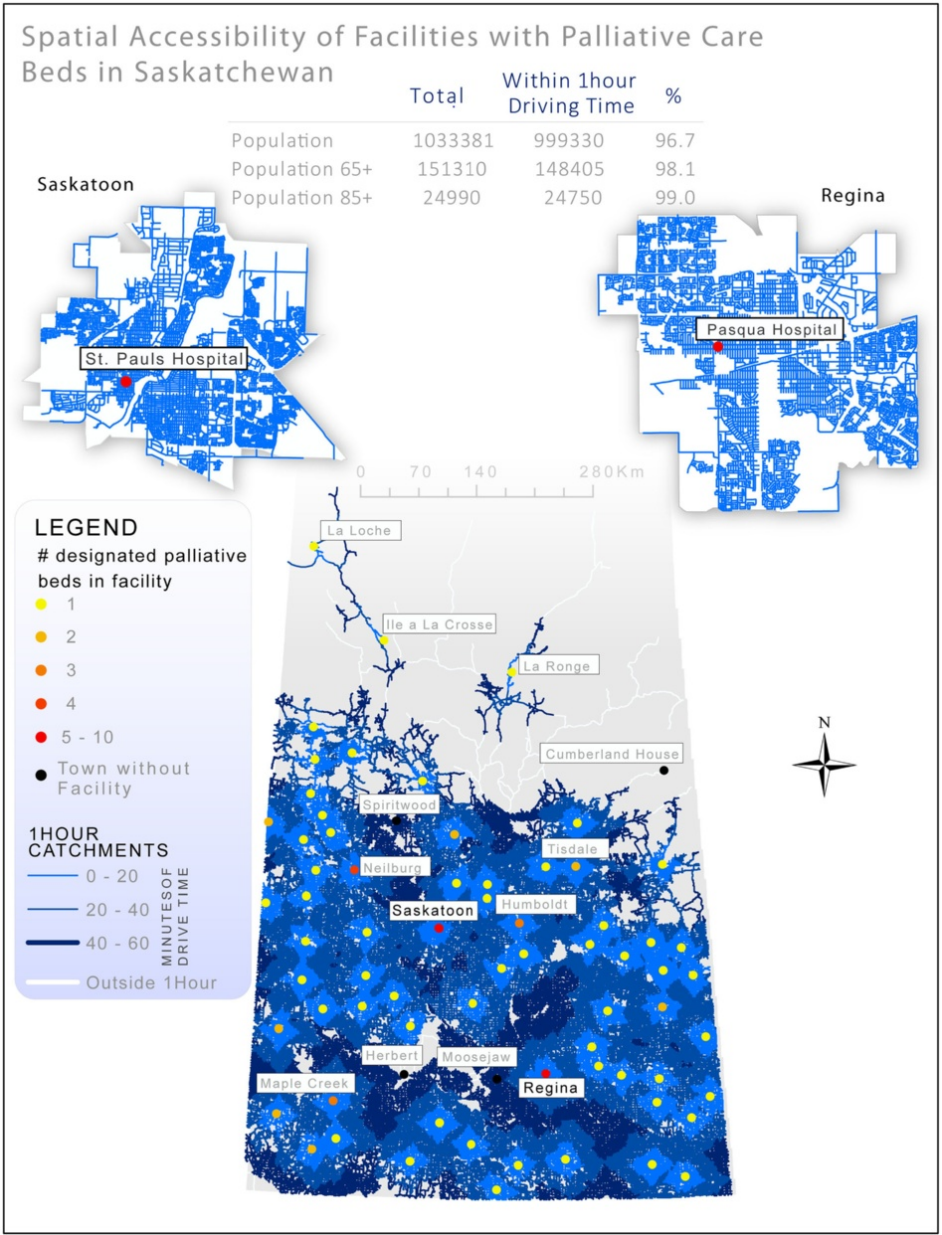
Illustrates Saskatchewan’s distinct pattern of palliative service provision with services available throughout the Southern populated areas of the province. This pattern of service provision is related to both topography (flat prairie) as well a tradition of supporting small rural hospitals – rather than centralizing services

In Saskatchewan, the land is unvaried with largely flat, grain growing plains in every direction making road travel relatively simple. Historically, land was distributed based on the Dominion Land Survey in regular one-square-mile ‘sections’. This led to extremely regular but entirely dispersed settlement and to an emphasis on maintaining generalist services in small rural hospitals. Under the leadership of Tommy Douglas (premier of Saskatchewan), the implementation of the Saskatchewan Hospital Services Plan in 1947 brought the expansion of hospital construction at three times the national average, with rural hospitals proliferating [21]; this has resulted in relatively good service provision in rural areas, albeit the recent hospital closures experienced in the last while [22]. This pattern of generalized, even spatial access to services throughout the province that results in 96.7 % of the population living within at most 1 h of drive time to a designated palliative care bed is illustrated in Fig. 2.

Newfoundland’s early settlement pattern was based on its reliance on fishing. As a resource based economy, settlement was confined to coastal areas with maximum dispersion along the rugged coastline in order to maintain access to the primary resource for small pockets of settler families. In many cases, merchant ships picked up the catch once or twice a year from coastal communities. As a result, many never left their coastal home. A program of forced relocation attempted to centralize settlement in the province but many coastal or outport communities remain and roads are not dense [23]. Since the province joined Canada in 1949, there have been some attempts to centralize services, such as the aggregation of hospitals [24], but the historical pattern of chiefly coastal settlement seems to remain the primary influence on where people live and, ultimately, where facilities with dedicated palliative care beds are located.

**Fig. 3:**
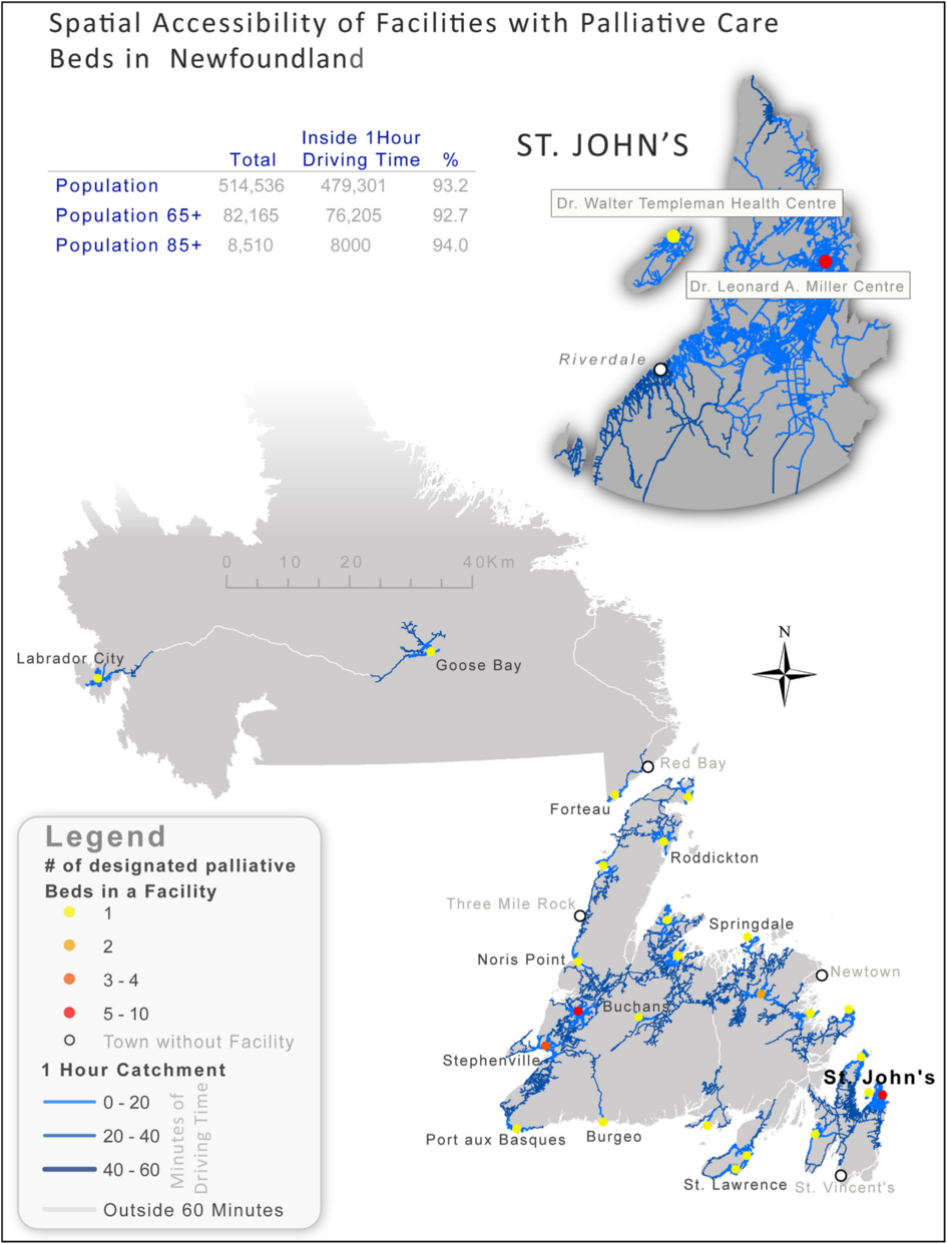
Illustrates that coastal settlements in Newfoundland are the nodes for facilities. This is distinct from the pattern in Saskatchewan where hospitals are regularly located through the heart of the province. This is a legacy of sea-based settlement – based on a fishing economy with historically limited access to the interior

Figure 3 illustrates another distinctive pattern with coastal settlements serving as the locus points for facilities with dedicated palliative care beds. Here we see the legacies of coastal settlement where ocean-going ships were the primary modes of transportation. Even though most of Newfoundland can now be traversed by automobile, the pattern of settlement – and access to palliative services – remains.

Potential spatial access alone cannot fully account for understanding the availability of dedicated palliative care beds in Newfoundland and Saskatchewan. These beds are often at a premium and lack of available beds can effectively limit temporal access for palliative patients. However, when the relationship between wait time and bed availability was examined for each facility within these two provinces, the relationship was found to be weak in Newfoundland (R^2^ = 0.26) and virtually nonexistent in Saskatchewan (R^2^ = 0.01). This is likely due to the fact that wait time is impacted not only by the number of beds available but also by the length of time a palliative patient will occupy a bed. The relationship may also be affected by the fact that some facilities convert regular beds to palliative beds as required. This flexibility provides the ability to prioritize palliative patients when necessary.

**Fig. 4:**
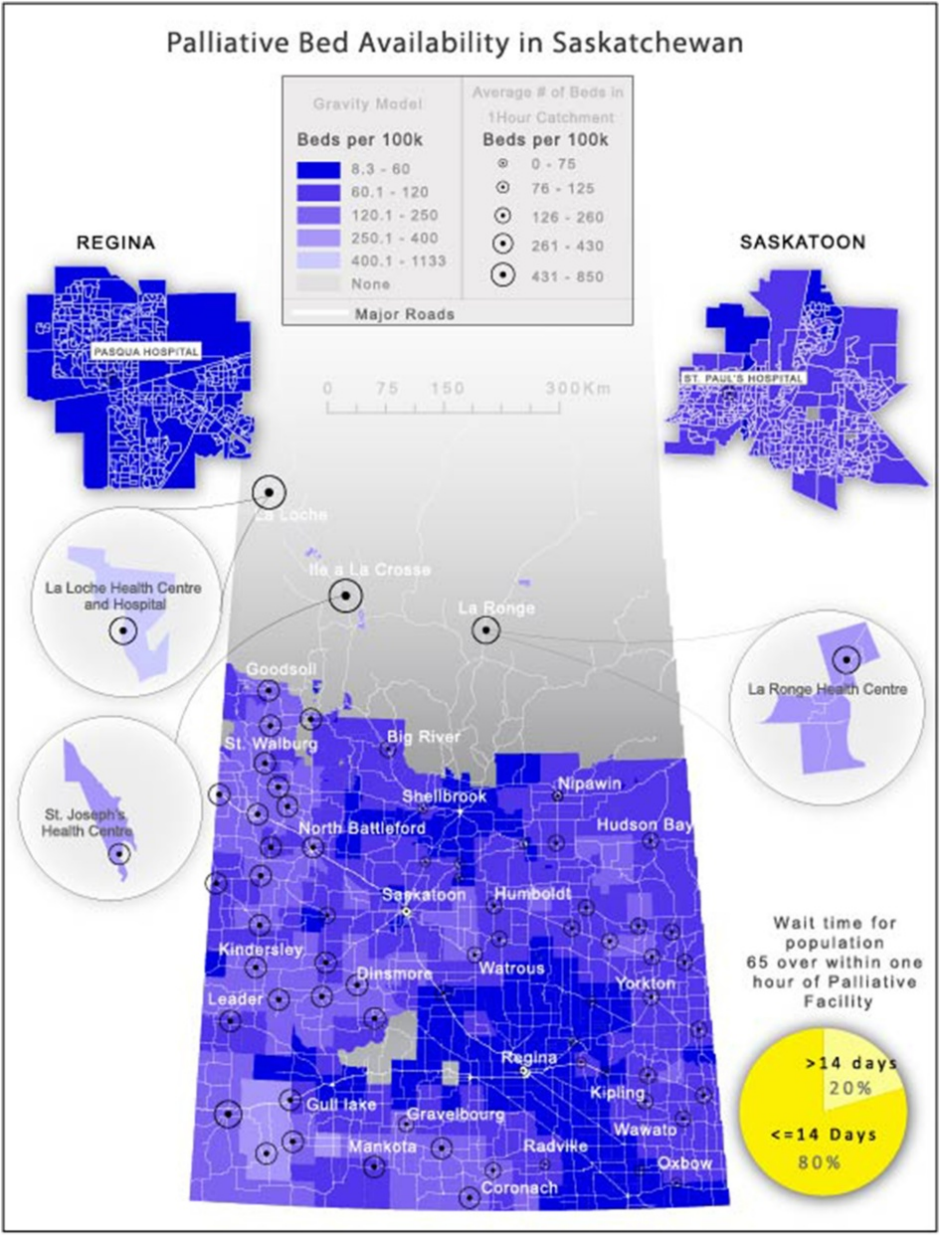
Illustrates that spatial access remains the most important determinant of access in Saskatchewan. Wait time is not perceived as a significant deterrent to access according to Health Regions surveyed for this study

Palliative care is affected not only by geography but by wait time. Wait time is a complex variable as end-of-life is unpredictable. Moreover, in non-tertiary hospitals, beds are often ‘mixed use’ and not exclusively designated for palliative use. In Saskatchewan, wait time is not such a critical factor in estimating access to palliative care. This is illustrated in Fig. 4 where we see that there is no correlation between wait time and bed availability. However, it should be noted that relatively accessible palliative care beds in Saskatchewan include both officially designated/funded palliative beds and the ad-hoc designations in smaller facilities.

**Fig. 5:**
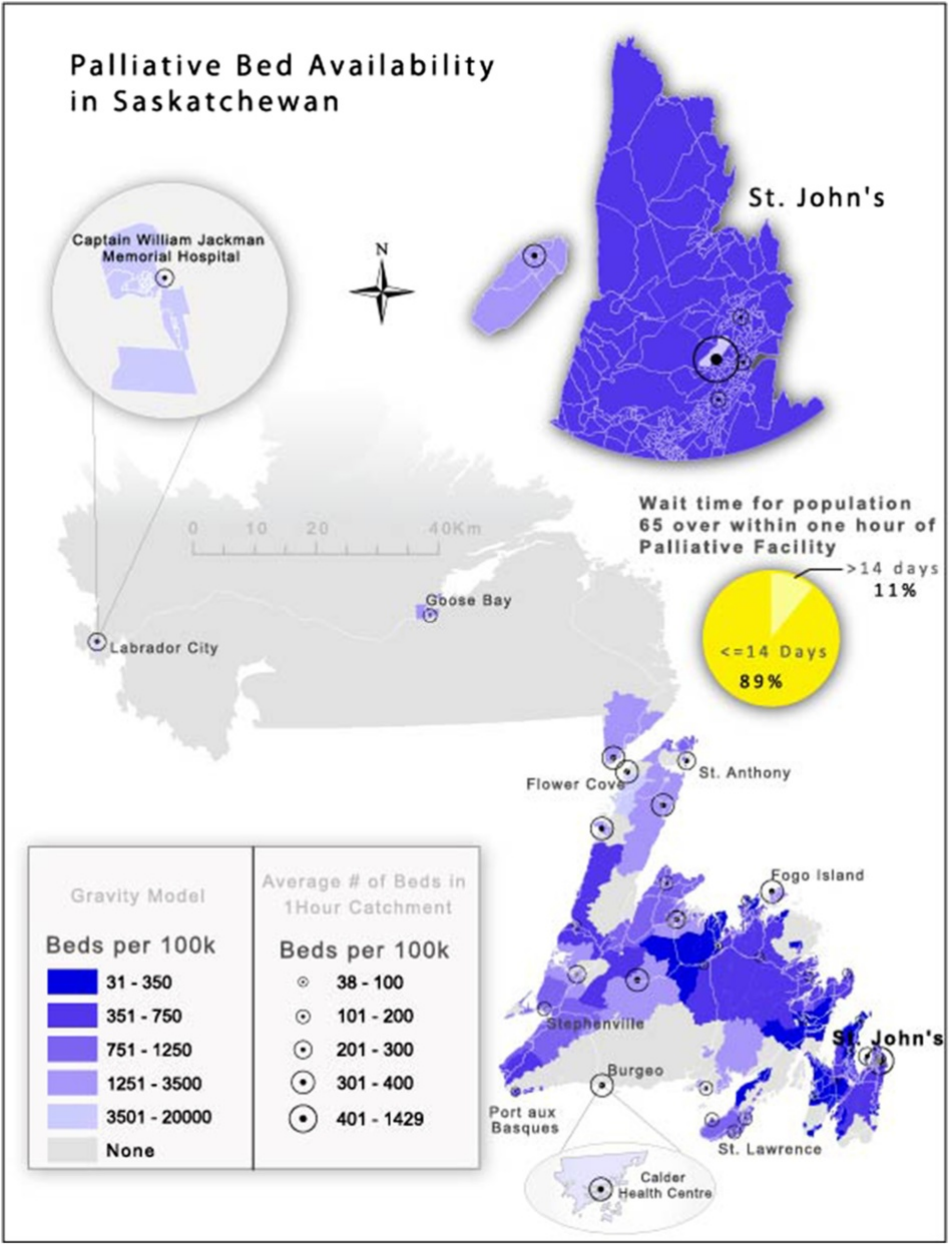
Though wait times in Newfoundland are only about 10 % higher than in Saskatchewan, combined with poor potential spatial access, they are a significant confounder with respect to access to palliative care

In Newfoundland wait time is a significant impediment to access to palliative care. In Fig. 5, we see clearly that wait time is a more problematic there than in Saskatchewan. In Newfoundland, access to dedicated palliative care beds clearly combines with spatial distance to confound overall access to palliative care services for residents in need. For example, the average wait time is approximately 4 weeks for those aged 65 and over. However, it is only when access (travel times) are combined with wait times that availability can be fully evaluated.

## Discussion and conclusion

The findings shared above show the spatial or geographical implications of health care decision-making and restructuring as they play out over time. For instance, Saskatchewan has closed some small hospitals [22] but it has kept a sufficient number of facilities open so that close to 100 % of its rural population can access a facility with a dedicated palliative care bed within a reasonable travel time (i.e., one hour). Newfoundland has a unique settlement pattern in its coastal communities relative to the rest of Canada, enjoying relatively good potential spatial access to such beds when compared to similarly sized towns in many provinces. However, the potential spatial access to these beds is not as complete as that provided in Saskatchewan.

Our spatial analysis shows that when wait times are incorporated as a way to understand potential *spatio-temporal* access to dedicated palliative care beds, as opposed to spatial access alone, the picture of access changes. In Saskatchewan, wait times are minimal and are thus not likely to affect realized access to a designated palliative care bed. In Newfoundland, however, wait times act as an additional barrier to realizing access to designated palliative care beds. Our development of an analytic technique for considering spatial *and* temporal access simultaneously using GIS methods demonstrates the potential for using these methods to generate new insights into a range of problems surrounding palliative care distribution and utilization. In our case, we used wait times (measured in weeks) combined with questions about the effects of wait times on availability (measured using a Likert scale) to create a picture of temporal impediments to care.

Moreover, this is an example of successfully implementing a mixed methods approach with qualitative assessments and explanations of palliative capacity interwoven with quantitative analyses of access. Our hope is that such information will be useful for health care services planning, in order to provide reasonable and equitable access to PCS. Further, the analytical methods employed herein can be applied to other health service allocation issues, such as access to mental health services.

This paper has demonstrated significant differences in potential spatio-temporal access to dedicated palliative care beds across two sample Canadian provinces. In each case, a combination of topography, historical settlement patterns and health allocation decision-making have combined to produce unique patterns of access to this end-of-life care. Moreover, the effects of wait times in confounding potential access are illustrated. Finally, the paper serves to illustrate the value of using GIS and spatial analysis to highlight distinct patterns of potential spatial-temporal access to services.

These analyses and comparisons are transferable to other jurisdictions. The requirement is GIS capacity with basic road travel time data as well as survey data about accessibility, beds and wait times. Such inter-jurisdictional comparisons are a means of independently evaluating relative access to care along multiple axes. The analysis provides a means of uncovering both physical and infrastructural constraints to seeking end-of-life care. However, the study also has several limitations. First, the study relied upon subjectively assessed wait times for palliative beds by relevant health workers rather than drawing from objectively recorded wait time counts. However, as the majority of the facilities in this analysis rely upon informal palliative capacity (in the sense they are not formally designated and funded as palliative-only beds), these assessments of palliative wait times are not available elsewhere. This was also a confounding factor in assessing the relationship between wait times and beds as “flex” beds can be occupied and thus give the impression of bed availability and long wait times. Secondly, while our survey collected a comprehensive account of additional palliative services and specialists at each site (e.g. music therapy, bereavement services, pain management), this analysis’ focus on wait times and spatial accessibility does not readily lend itself to incorporating these additional data.

No reliable data specific to dedicated palliative care bed wait times are kept nationally, provincially, or regionally within Canada. Because of this we had to create our own subjective indicator. Specifically, we called each facility within the provinces of focus and asked to speak with the person most knowledgeable about palliative care bed demand and availability. We administered our phone survey to this individual, using the same wording each time to ensure comparability across sites. A limitation of this approach is that we were gathering perceptual insights regarding wait times on a Likert scale rather than some other more rigorous form of measurement, but a strength is that we were able to capture the first insights into dedicated palliative care bed wait times in these facilities and across provinces in a manner that was acceptable to all the local health authority research ethics boards that had to approve our protocol.

Lastly, the study overlooked the provision of palliative care services within the home, even though such services form a large part of the palliative support provided within each province. As this data is not readily available, let alone standardized across provincial boundaries, and the focus of our study is on institutional palliative capacity, it is beyond the scope of this analysis. Despite the noted limitations, this analysis provides the basis for more structured and granular comparisons of palliative facilities.

## Abbreviations

PCS: Palliative care services
DA: Dissemination Area

## Footnotes

1 By ‘dedicated palliative care bed’ we refer to beds at inpatient medical facilities that are prioritized for use by palliative care recipients. In large hospitals in the provinces of focus these beds tend to be used only by palliative care recipients, and were sometimes located in palliative care wards. In small hospitals these were usually beds located in private rooms that could accommodate family visits and were primarily, but not exclusively, used by palliative care recipients.
